# Knowledge, Attitudes, and Smoking Behaviours among Physicians Specializing in Public Health: A Multicentre Study

**DOI:** 10.1155/2014/516734

**Published:** 2014-06-03

**Authors:** Giuseppe La Torre, Rosella Saulle, Brigid Unim, Italo Francesco Angelillo, Vincenzo Baldo, Margherita Bergomi, Paolo Cacciari, Silvana Castaldi, Giuseppe Del Corno, Francesco Di Stanislao, Augusto Panà, Pasquale Gregorio, Orazio Claudio Grillo, Paolo Grossi, Francesco La Rosa, Nicola Nante, Maria Pavia, Gabriele Pelissero, Michele Quarto, Walter Ricciardi, Gabriele Romano, Francesco Saverio Schioppa, Roberto Fallico, Roberta Siliquini, Maria Triassi, Francesco Vitale, Antonio Boccia

**Affiliations:** ^1^Department of Public Health and Infectious Diseases, Sapienza University of Rome, Piazzale Aldo Moro 5, 00185 Rome, Italy; ^2^Department of Experimental Medicine, Second University of Naples, Via Luciano Armanni 5, 80138 Naples, Italy; ^3^Department of Environmental and Public Health, University of Padova, Via Giustiniani 2, 35128 Padova, Italy; ^4^Department of Science of Public Health, University of Modena e Reggio Emilia, Via Campi 287, 41100 Modena, Italy; ^5^Department of Medicine and Public Health, University of Bologna, Via S. Giacomo 12, 40126 Bologna, Italy; ^6^Department of Medicine and Public Health, Virology, University of Milano, Via Pascal 38, 20133 Milan, Italy; ^7^Department of Clinical Medicine and Prevention, University of Milano Bicocca, Via Cadore, 48, 20052 Monza, Italy; ^8^Department of Biomedicine Science, Section of Hygiene, Preventive Medicine and Public Health, University of Ancona, Via Tronto 10/a, 60020 Ancona, University of Ancona, Italy; ^9^Department of Public Health, University of Tor Vergata, Rome, Via Orazio Raimondo 18, 00173 Rome, Rome, Italy; ^10^Department of Clinical and Experimental Medicine, University of Ferrara, Via Fossato di Mortara 64b, 44121 Ferrara, Italy; ^11^Department of Hygiene, Preventive Medicine and Public Health (R. De Blasi), University of Messina, Italy; ^12^Department of Clinical Medicine, University of Varese, Insubria, Via Ravasi 2, 21100 Varese, Italy; ^13^Department of Public Health, University of Perugia, Via Roma 84, 06019 Perugia, Italy; ^14^Department of Physiopathology, Experimental Medicine and Public Health, University of Siena, Via Aldo Moro 2, 53100 Siena, Italy; ^15^Department of Experimental and Clinical Medicine (Gaetano Salvatore), University of Catanzaro, Via T. Campanella 115, 88100 Catanzaro, Italy; ^16^Department of Preventive Medicine-Section of Hygiene, University of Pavia, Via Forlanini 2, 27100 Pavia, Italy; ^17^Section of Hygiene, Department of Biomedical Science and Human Oncology, University of Bari, Piazza Giulio Cesare 11, 70124 Bari, Italy; ^18^Institute of Hygiene, University “Cattolica del Sacro Cuore” of Rome, Via Ippolito Nievo 36, 00153 Rome, Italy; ^19^Section of Hygiene and Preventive Medicine, Department of Public Health and Community Medicine, Via dell'Artigliere 8, 31129 Verona, University of Verona, Italy; ^20^Department of Medicine and Aging Science, University “G. Annunzio” Chieti, Via dei Vestini 33, 66100 Chieti, Italy; ^21^Department “G. F. Ingrassia” Hygiene and Public Health, University of Catania, Via S. Sofia 78, 95123 Catania, Italy; ^22^Department of Public Health and Microbiology, University of Turin, Via Santena 5 bis, 10126 Torino, Italy; ^23^Section of Hygiene, Department of Science Preventive Medicine, University of Napoli Federico II, Via Tommaso Campanella 115, 88100 Napoli, Italy; ^24^Department of Health Promotion Science (G. D'Alessandro), University of Palermo, Via Del Vespro 133, 90127 Palermo, Italy

## Abstract

*Background*. Healthcare professionals have an important role to play both as advisers—influencing smoking cessation—and as role models. However, many of them continue to smoke. The aims of this study were to examine smoking prevalence, knowledge, attitudes, and behaviours among four cohorts physicians specializing in public health, according to the Global Health Profession Students Survey (GHPSS) approach. *Materials and Methods*. A multicentre cross-sectional study was carried out in 24 Italian schools of public health. The survey was conducted between January and April 2012 and it was carried out a census of students in the selected schools for each years of course (from first to fourth year of attendance), therefore among four cohorts of physicians specializing in Public Health (for a total of n. 459 medical doctors). The GHPSS questionnaires were self-administered via a special website which is created ad hoc for the survey. Logistic regression model was used to identify possible associations with tobacco smoking status. Hosmer-Lemeshow test was performed. The level of significance was *P* ≤ 0.05. *Results*. A total of 388 answered the questionnaire on the website (85%), of which 81 (20.9%) declared to be smokers, 309 (79.6%) considered health professionals as behavioural models for patients, and 375 (96.6%) affirmed that health professionals have a role in giving advice or information about smoking cessation. Although 388 (89.7%) heard about smoking related issues during undergraduate courses, only 17% received specific smoking cessation training during specialization. *Conclusions*. The present study highlights the importance of focusing attention on smoking cessation training, given the high prevalence of smokers among physicians specializing in public health, their key role both as advisers and behavioural models, and the limited tobacco training offered in public health schools.

## 1. Introduction

Tobacco use continues to be the leading cause of preventable disease and it is responsible for more than 5 million deaths 3 each year worldwide [1].

Despite this, there are still 650 million smokers in the world. In Italy, the prevalence of smoking among adults was 22.7% in 2011, and cigarette smoking accounts for approximately 25% deaths annually. Prior to the introduction of the law in March 2003 banning smoking in all indoor public places, the prevalence was 23.8% [1]. Undoubtedly, there has been a gradual reduction in smoking prevalence in the last years in Italy and in other western countries, but smoking remains the main cause of mortality and morbidity [1].

Tobacco dependence has actually many aspects of a chronic disease: most patients do not achieve abstinence after their first attempt to quit, they have periods of relapse and they often require repeated cessation interventions [2].

Unfortunately, the percentage of smokers who seek help from physicians to quit smoking has reduced over years in Italy (6.2% in 2009 versus 3.6% in 2011), and also the number of quitting attempts (30.5% in 2007 versus 26.7% in 2011) [3].

High rates of smoking among doctors and other health care workers (HCW) and limited training on cessation approaches may compromise the ability of physicians to effectively treat their patients who smoke. In fact, tobacco dependence counselling in medical schools are scarce, as indicated in recent surveys [4, 5].

Several studies have demonstrated the efficacy of smoking cessation programs and the importance of physician's advice to their patients [6, 7].

Most randomized controlled trials (RCTs) report a cessation success ranging from 15 to 35% after six or twelve months of followup [7]. Generally, the available pharmacological treatments and the group/individual counselling are effective in smoking cessation, though integrated tobacco cessation programs and services usually give higher percentages of success [8].

There is evidence that in Italy physicians are not advising smokers to quit [9, 10]; nevertheless HCW have an important role to play both as advisers—influencing smoking cessation—and as role models and [11, 12] HCW, and particularly physicians, should be encouraged to assist smokers to quit, especially considering that almost half of former smokers indicate health conditions as the main reason to stop smoking [13].

The smoking habits of medical students—especially of physicians specializing in public health—have only rarely been the object of studies and interventions in Italy, and the focus of the published literature is generally narrow. For these reasons, we decided to carry out a nationwide survey of physicians specializing in public health in order to describe and analyze the smoking habits of this population.

The aims of the present study are as follows:

- to evaluate smoking prevalence, knowledge and attitudes, and tobacco cessation training among public health resident physicians in Italy;
- to examine the difference between smokers and nonsmokers;
- to estimate the extent of teaching about tobacco and smoking cessation techniques in public health schools in Italy;
- to recommend the integration of tobacco-related education in the curriculum of future public health professionals.

## 2. Materials and Methods

In this multicentre cross-sectional study, 24 Italian schools of public health—from a total of 27 Italian schools—were involved (Catania, Bologna, Bari, Catanzaro, Siena, Chieti, Messina, Modena, Ferrara, Ancona, Milano, Pavia, Verona, Perugia, Palermo, Roma Sapienza, Roma Tor Vergata, Roma Cattolica, Milano Bicocca, Torino, Napoli, Napoli Federico II, Insubria, and Padova).

The survey was conducted between January and April 2012. The study was made possible by the school councils of each university: we contacted each director of all the 27 Italian public health schools (n. 531 medical doctors specializing in public health) and 24 Schools approved the survey (88.9%). So it was carried out a census of students in the selected schools for each years of course (from first to fourth year of attendance), therefore among four cohorts of physicians specializing in Public Health (for a total of n. 459 medical doctors). The directors of each public health school organized the meeting with representatives of medical doctors specializing in public health who assisted us in communicating and informing all colleagues about the survey and the questionnaire administration via web; they also solicited them to complete the questionnaire for the survey.

Resident physicians' representatives, prior to the survey, were informed through a detailed letter via mail about the code assignment mechanism. Their duty was to assign a single code, for the GHPSS website, to each medical doctor. The codes were different to enable the identification of the 24 schools, the year of attendance and the number of MDSPH for each year of course. A total of 388, out of 459 medical doctors, participated in the survey on the website (85% response rate).

The questionnaires were administered with close-ended type of questions with more response options, in an anonymous, voluntary manner, in accordance with the protocol developed by WHO Europe and the US CDC [14].

The original questionnaire was composed of 42 questions divided into six sections, but in the current study we added one country-specific question on knowledge about the use of antidepressants (such as bupropion or Zyban) and acetylcholine receptor partial agonists (such as Varenicline or Champix) and counselling techniques in tobacco cessation programs.

The final form of the Italian questionnaire was composed of 44 questions, distributed in 6 sections on the following:

1. prevalence of tobacco use (Questions 1–9);
2. exposure to environmental tobacco smoke (i.e., time spent with people who smoke in places other than home) (Questions 10–13);
3. attitudes (i.e., opinions about no-smoking policies and laws, and about the role of healthcare professionals in smoking cessation) (Questions 14–24);
4. behavior/cessation (i.e., smoking habit, willingness to stop, and opinions about healthcare professionals who used to smoke) (Questions 25–32);
5. curriculum/training (i.e., formal training in smoking cessation techniques on the medical curriculum and knowledge about methods (pharmacological or counseling techniques) for helping smokers to quit) (Questions 33–41—in the original version previous adding the two new therapies. So in the new version the 5 section resulted form 33–41); and
6. demographics (age, gender, and course year) (Questions 42–44).

All items were considered and analyzed, but here we reported only the significant results. Our attention was focused in particular on questions about smoking behavior and intention to quit, attitudes regarding the role of healthcare professionals in smoking cessation, and training and knowledge about smoking cessation methods.

We were able to enter every time in the data base of the website to check the completed questionnaires and after two month from the start of the survey, we send a new letter via mail to each representative of the school to solicit all colleagues to complete the questionnaire.

### 2.1. Outcome Measure

The outcome measure was “being a current smoker”—who smoked cigarettes at least 1 day during the 30 days before the survey (WHO 2010)—in the four cohorts of physicians specializing in public health.

### 2.2. Statistical Analysis

Data were analysed with the software SPSS 19.0 for Windows.

Descriptive analyses were performed using frequencies, percentages, frequency tables for categorical variables and mean ± standard deviation (SD), and 95% confidence intervals (95% CI) for quantitative variables.

Chi-square tests were performed to evaluate differences for categorical variables. A logistic regression model was used to identify possible factors associated with the tobacco smoking status. According to the Hosmer-Lemeshow procedure, only covariates having a *P* value <0.25 at univariate analysis were introduced into the models [15].

Moreover, gender and age, as possible confounders, were included into the regression model.

Before the analysis all variables were transformed into binary ones. Results are expressed as odds ratio (OR) with 95% CI, and the goodness of fit of the model was assessed by the Hosmer-Lemeshow test. The level of significance was set at *P* ≤ 0.05.

## 3. Results

The prevalence of current smokers was 20.9% (n. 81), about 73% have smoked at least once in their life and the age of cigarette initiation was 16-17 years for 25.8% of the sample. Among current smokers, 26.2% were males versus 73.8% that were nonsmokers (*P* ≤ 0.001), 16.2% of the smokers were females versus 83.8% that were nonsmokers; about 55.6% of the total smokers was over 30 years (*P* = 0.1).

Sociodemographic characteristics are shown in Table 1.

The first cohort (1° year of course) had the highest percentage of smokers (29.6%), while the third cohort (3° year of course) presented the lowest rate (18.9%) (*P* = 0.01). Attitudes, beliefs, and knowledge about tobacco are reported in Table 2.

Health professionals should receive specific training on smoking cessation according to 93% of the sample, while 5.7% were of the opposite opinion. HCW represent a role model for their patients and the general population for 80% of physicians, and 98% declared that HCW have an important role in advising patients to quit smoking. In addition, 87% of attendants affirmed that a patient has more probability to quit smoking if assisted by a HCW.

Relatively to knowledge on tobacco related issues (Table 2), many responders followed lessons on smoking risk during their postgraduate course (67.4% nonsmokers and 76% current smokers) but specific training on smoking cessation was given to only 17% of physicians during their specialization.

Contrarily, 388 (89.7%) responders have heard about smoking issues during their undergraduate courses. Furthermore, most participants have heard about nicotine patches or gum (97%) and 43% knew about antidepressant, such as Bupropion or Zyban, used in cessation programs. Less than 50% of participants have been taught about the importance of patients smoking history as part of anamnesis and about distributing informative materials on smoking cessation to patients.

Multivariate analysis for the outcome “being current smokers” showed that males have significantly an higher odds to be smokers in comparison to females (OR = 1.83, 13 95% CI: 1.09–3.07; adjusted OR = 1.79%, CI: 1.06–3.03) and medical doctor specializing from South Universities were significantly more likely to be smokers in comparison to the Centre Universities (south: OR = 1.67, 95% CI: 0.99–2.87; adjusted OR = 1.85, 95% CI: 1.05–3.24) while no significant differences resulted in relation to the North Universities in comparison to the centre ones (north: OR = 0.93, 95% CI: 0.57–1.52; adjusted OR = 1.51, 95% CI: 0.77–2.95) (Table 3).

## 4. Discussion

Health professionals have an important role in providing evidence-based tobacco interventions for both smoking cessation and prevention. However, limited and inconsistent levels of tobacco training are currently being provided to health care students [8, 16–19].

One of the aims of this study was to assess the tobacco-related education currently offered in Italian Public health schools.

The results indicate that there is minimal education about smoking related-issues in public health programs. In particular, only 17% of the sample received specific training on smoking cessation, up to 60% have never heard about pharmacological treatments used for quitting interventions and have never been taught about the psychological factors influencing tobacco use.

The importance of patients smoking history or giving informative materials to help patients quit smoking is still unknown to most medical doctors, though most participants have followed lessons on smoking risk during their undergraduate and postgraduate courses.

According to the “Clinical Practice Guideline for Treating Tobacco Use and Dependence” of the US Public Health Service (PHS), to increase tobacco cessation rates with the counseling and with any of the seven Food and Drug Administration (FDA) approved first-line medications. Exceptions are made for medically contraindicated populations or when evidence of effectiveness is insufficient, such as pregnant women, smokeless tobacco users, light smokers, and adolescents [20].

Unfortunately, a study carried out by Ferketich et al. [21] found a low prevalence of ascertainment of smoking status, documentation of tobacco cessation assistance among tobacco users, and pharmacotherapy prescription.

The percentage of smokers in the sample (20.9%) is quite high considering their medical background; a much lower rate of current smokers among resident physicians was expected compared to the Italian national rate of 22.7% in 2011 [6].

Similar findings are reported in the study by Saulle et al., 2013, conducted with the GHPSS approach and regarding third year medical students [22]. In particular, the prevalence of smokers (20.4%) is high and the majority of the students declared that health professionals have an important role in smoking cessation process (65%), and need specific training (87.7%). Moreover, 89.4% have not received specific training on smoking cessation techniques and students belonging to universities in southern Italy were more likely to be smokers, as in the present study. The paper by Saulle et al. confirms the results of the present work regarding lack of specific training offered to future health professionals and the major probability of being a smoker in Southern Universities.

About 69% of current smokers have smoked on school premises/property during the past year despite 92% of the sample reported that their university had a ban on smoking in all school buildings and clinics. These aspects of the participants could be attributed to lack of knowledge on smoking issues.

Implementing teaching about tobacco in the medical curricula could reduce the smoking prevalence among MDSPH and increase the possibility that they deliver information about the health effects of smoking and influence smokers [2, 23].

There are some limitations of this survey that must be stressed. Firstly, the study has a cross sectional design with self-reported data that could lead to underreporting and recall bias. In addition, a very strict definition of smoking was used, based on WHO's standard definition for smoking status. However, a validated questionnaire was used.

The validation of GHPSS survey in Italy was carried out by “Sapienza” University and the Catholic University of Sacred Heart in Rome [24, 25].

The original survey questionnaire, developed by WHO, the US CDC, and the CPHA, is a standard pretested questionnaire for assessing prevalence of tobacco use among health care professionals around the world [26].

The strengths of this study are high response rate (85%) and strong level of significance of several results. To our knowledge, this is the first Italian survey regarding resident physicians in public health carried out according to the GHPSS methodology.

Health care professionals have the unique opportunity to affect tobacco-related morbidity and mortality. Therefore, adequate training of medical doctors is crucial to promote cessation among tobacco users and provide effective public health service in terms of counselling and medication. Learning of epidemiological aspects of smoking in youngsters, especially medical students, is of great importance for the whole community. The smoking habits of medical students are affected by the same phenomena that affect those of the general public, such as the influence of socio cultural factors and the increasingly broader age range of initiation and other similar influence.

## 5. Conclusion

All healthcare professionals play an important role in the process of smoking cessation both as advisers and behavioural models for the general population. Regarding physicians specializing in public health, the prevalence of smokers is high (over 20%) and alarming considering their key role in public health interventions for the promotion of healthy lifestyles. In the field of public health, tobacco screening, and intervention is one of the most effective clinical preventive services [5, 27]. Planning and implementing smoking cessation training and cessation tailored to these young health professionals is therefore strongly recommended.

## Figures and Tables

**Table 1:** Characteristics of the sample.

Sociodemographic variables	Frequencies *N* (%)	Nonsmokers *N* (%row)	Current smokers *N* (%row)	*P* value
Age				
<30	141 (36.3)	105 (74.5)	36 (25.5)	0.1^∧^
≥30	247 (63.7)	202 (81.8)	45 (18.22)	
Gender				
F	247 (63.7)	207 (83.8)	40 (16.2)	0.02^∗∧^
M	130 (34.5)	96 (73.8)	34 (26.2)	
Year of attendance				
1st	104 (27.6)	80 (76.9)	24 (23.1)	
2nd	101 (26.8)	82 (81.2)	19 (18.8)	0.7^∧^
3rd	88 (23.3)	74 (84.1)	14 (15.9)	
4th	84 (22.3)	67 (79.8)	17 (20.2)	
Macroarea				
North	178 (45.9)	142 (79.8)	36 (20.2)	
Centre	113 (29.1)	95 (84.1)	18 (15.9)	0.1^∧^
South	97 (25)	70 (72.2)	27 (27.8)	

Total	388 (100)		81 (20.9)	

*P < 0.05 (level of significance); ^∧^ *P* value concerns the difference between smokers and nonsmokers for each sociodemographic variable considered.

**Table 2:** Health care providers' role in cessation (in smokers and nonsmokers sample).

Opinions on health care providers' role in smoking cessation	Frequencies *N* (%)^a^	Nonsmokers *N* (%)^b^	Current smokers *N* (%)^c^	*P* value
Should HPs get specific training on cessation techniques?				
Yes	361 (94.3)	291 (95.1)	70 (90.9)	
No	22 (5.7)	15 (4.9)	7 (9.1)	0.002^∗∧^
HPs serve as role models for their patients and the public?				
Yes	309 (80.7)	252 (82.4)	57 (74.0)	
No	74 (19.3)	54 (17.6)	20 (26.0)	<0.001^∗∧^
Should HPs regularly advise smokers to quit?				
Yes	377 (98.4)	303 (99.0)	74 (96.1)	
No	6 (1.6)	3 (1.0)	3 (3.9)	0.001^∗°∧^
Should HPs regularly advise smokers to quit chewing tobacco/smoking cigar or pipe?				
Yes	373 (97.4)	299 (97.7)	74 (96.1)	0.003^∗°∧^
No	10 (2.6)	7 (2.3)	3 (3.9)	
Do HPs have a role in giving advice or information about smoking cessation to patients?				
Yes	375 (97.9)	299 (97.7)	76 (98.7)	0.004^∗°∧^
No	8 (2.1)	7 (2.3)	1 (1.3)	
Patients have more chances to quit smoking if helped by HPs?				
Yes	332 (86.7)	272 (88.9)	60 (77.9)	<0.001^∗∧^
No	51 (13.3)	34 (11.1)	17 (22.1)	

Postgraduate tobacco-related training	Frequencies *N* (%)^d^	Nonsmokers *N* (%)^e^	Current smokers *N* (%)^f^	*P* value

Have you been taught about smoking risk during your postgraduate course?				
Yes	262 (69.1)	205 (67.4)	57 (76.0)	0.001^∗∧^
No	117 (30.9)	99 (32.6)	18 (24.0)	
Have you ever receive specific training on smoking cessation during your postgraduate course?				
Yes	66 (17)	53 (17.3)	13 (16)	0.003^∗∧^
No	313 (80.7)	251 (81.8)	62 (76.5)	
Have you ever heard, during your postgraduate course, about nicotin patches or gum used in cessation programs?				
Yes	368 (94.8)	294 (95.8)	74 (91.4)	0.002^∗∧^
No	11 (2.8)	10 (3.3)	1 (1.2)	
Have you ever heard, during your specialization, about antidepressant (Bupropion or Zyban) used in cessation programs?				
Yes	163 (43.0)	129 (42.4)	4 (45.3)	0.003^∗∧^
No	216 (57.0)	175 (57.6)	41 (54.6)	
Have you been taught about the importance of providing informative materials to help patients quit smoking?				
Yes	195 (51.5)	154 (50.6)	41 (54.6)	0.002^∗∧^
No	184 (48.5)	150 (49.3)	34 (45.3)	
Have you been taught about the importance of registering patients smoking history as part of the anamnesis?				
Yes	222 (58.6)	183 (60.2)	39 (52.0)	0.001^∗∧^
No	157 (41.4)	121 (39.8)	36 (48.0)	

^a^5 missing values for “attitude and beliefs”; ^b^1 missing value for “attitude and beliefs” among nonsmokers; ^c^4 missing values for “attitude and beliefs” among current smokers;^ d^9 missing values for variables concerning knowledge; ^e^3 missing values for nonsmokers; ^f^6 missing values among current smokers.

Chi-square test was performed to evaluate differences for categorical variables.

^∧^ *P* value is the difference between smokers and nonsmokers in each variable considered.

HPs: Health professionals; *P < 0.05 (level of significance); °Fisher's exact test.

**Table 3:** Results of the binary logistic regression analysis for the outcome “being current smokers.” Dependent variable: “being current smokers.” Independent variable: age, gender, year of attendance, macroregion (south), macro-region (north), macroregion (centre), “should HPs get specific training on cessation techniques?”; “do HPs serve as role models for their patients and the public?”; “should HPs regularly advise smokers to quit chewing tobacco/smoking cigar or pipe?”; “do HPs have a role in giving advice or information about smoking cessation to patients?”.

Variables	Crude OR (95% CI)	Adjusted OR (95% CI)
Age		
<30 (reference)*	1	1
≥30	0.65 (0.39–1.07)	0.88 (0.51–1.56)
Gender		
Female (reference)*	1	1
Male	1.83 (1.09–3.07)	1.79 (1.06–3.03)
Year of attendance**		
1-2 years (reference)*	1	1
3-4 years	1.05 (0.57–1.92)	1.06 (0.56–1.2)
Macroregion		
Centre (reference)*	1	1
South	1.67 (0.99–2.87)	1.85 (1.05–3.24)
Macroregion		
Centre (reference)*	1	1
North	0.93 (0.57–1.52)	1.51 (0.77–2.95)
Should HPs get specific training on cessation techniques?		
No (reference)*	1	1
Yes	0.51 (0.20–1.31)	0.64 (0.23–1.82)
Do HPs serve as role models for their patients and the public?		
No (reference)*	1	1
Yes	0.61 (0.34–1.1)	0.58 (0.32–1.05)
Should HPs regularly advise smokers to quit chewing tobacco/smoking cigar or pipe?		
No (reference)*	1	1
Yes	0.57 (0.14–2.29)	2.09 (0.2–22.77)
Do HPs have a role in giving advice or information about smoking cessation to patients?		
No (reference)*	1	1
Yes	0.24 (0.48–1.23)	0.31 (0.06–1.61)

Hosmer and Lemeshow Test: *P* = 0.62.

*Reference group; **Hosmer-Lemeshow procedure (Hosmer and Lemeshow 1989) [15] (only covariates having a *P* value <0.25 at univariate analysis were introduced into the models).

All variables were transformed into binary ones.

Age: <30 versus ≥30 (because the possible answers were 25–29 or ≥30); gender: female versus male; year of attendance: 1-2 years versus 3-4 years; macroregion: centre versus south and centre versus north; and all the others (should HPs get specific training on cessation techniques?; do HPs serve as role models for their patients and the public?; should HPs regularly advise smokers to quit chewing tobacco/smoking cigar or pipe?; do HPs have a role in giving advice or information about smoking cessation to patients?) as no versus yes.
